# Heat shock proteins HSPB8 and DNAJC5B have HCV antiviral activity

**DOI:** 10.1371/journal.pone.0188467

**Published:** 2017-11-28

**Authors:** Ana Claudia Silva Braga, Bruno Moreira Carneiro, Mariana Nogueira Batista, Mônica Mayumi Akinaga, Cíntia Bittar, Paula Rahal

**Affiliations:** 1 Laboratório de Estudos Genômicos, UNESP/IBILCE, São José do Rio Preto, São Paulo, Brazil; 2 Instituto de Ciências Exatas e Naturais, UFMT/CUR, Rondonópolis, Mato Grosso, Brazil; Saint Louis University, UNITED STATES

## Abstract

Hepatitis C is a disease caused by the hepatitis C virus (HCV), and an estimated 3% of the world population is infected with the virus. During replication, HCV interacts with several cellular proteins. Studies have shown that several heat shock proteins (HSPs) have an altered expression profile in the presence of the virus, and some HSPs interact directly with HCV proteins. In the present study, we evaluated the expression levels of heat shock proteins *in vitro* in the presence and absence of HCV. The differential expression of 84 HSPs and chaperones was observed using a qPCR array, comparing HCV uninfected and infected Huh7.5 cells. To validate qPCR array, the differentially expressed genes were tested by real-time PCR in three different HCV models: subgenomic HCV replicon cells (SGR-JFH-1), JFH-1 infected cells (both genotype 2a) and subgenomic S52 cells (genotype 3). The HSPB8 gene showed increased expression in all three viral models. We silenced HSPB8 expression and observed an increase in viral replication. In contrast, when we increased the expression of HSPB8, a decrease in the HCV replication rate was observed. The same procedure was adopted for DNAJC5B, and HCV showed a similar replication pattern as that observed for HSPB8. These results suggest that HSPB8 may act as an intracellular factor against hepatitis C virus replication and that DNAJC5B has the same function, with more relevant results for genotype 3. We also evaluated the direct interactions between HCV and HSP proteins, and the IP experiments showed that the HCV NS4B protein interacts with HSPB8. These results contribute to a better understanding of the mechanisms involved in HCV replication.

## Introduction

Hepatitis C is a disease caused by the hepatitis C virus (HCV), and an estimated 115 million people are infected with the virus worldwide [1]. Most patients are asymptomatic and are typically diagnosed only after a long period of time, resulting in complications, such as cirrhosis, hepatic failure and hepatocarcinoma [2].

HCV is an enveloped virus belonging to the family *Flaviviridae* in the genus *Hepacivirus* [3]. The HCV genome consists of a single-stranded, positive-sense RNA molecule that encodes a polyprotein precursor of approximately 3000 amino acids. This precursor is subsequently cleaved by cellular and viral proteases to generate structural proteins (core, E1, E2 and p7) and nonstructural proteins (NS2, NS3, NS4A, NS4B, NS5A, and NS5B)[4, 5].

The hepatitis C virus has six known genotypes, and a seventh has been reported [6, 7]. Genotypes 1, 2 and 3 are widely distributed throughout the USA, Europe, Australia and East Asia (Japan, Thailand and China). Genotype 4 is typically found in the Middle East, Egypt and Central Africa, and genotypes 5 and 6 are predominantly found in South Africa and Southeast Asia, respectively [8–10].

Several cellular proteins are known to interact with HCV or be necessary for the viral replication process [11]. Studies have shown that numerous heat shock proteins (HSPs) have altered expression profiles in the presence of the virus. Some HSPs interact directly with HCV proteins, especially because the virus does not have HSPs and depends on these host proteins for the correct folding of viral proteins[12, 13].

HSPB8 (or HSP22) belongs to the small heat shock family of proteins that are ubiquitously expressed in virtually all tissues [14]. HSPB8 prevents the accumulation of aggregated proteins in the cell and participates in the regulation of proteolysis of unfolded proteins [15]. Furthermore, HSPB8 appears to be directly or indirectly involved in regulation of apoptosis and carcinogenesis, contributes to cardiac cell hypertrophy and survival and, when mutated, may be involved in the development of neurodegenerative diseases [16–19].

Cysteine string proteins (CSPs) are a DnaJ chaperone family associated with regulated secretory organelles in organisms ranging from fruit flies to humans, and mammals have three CSP genes (α, β and γ) [20].

Viruses that promote chronic infection may change the expression of some HSPs to ensure a persistent viral infection[13]. The expression of the HSP70 protein is reduced in HCV infections and the HSP27 protein is overexpressed in cells containing a subgenomic replicon of HCV[21, 22]. HCV NS5A interacts weakly with HSPA5 and can alter the expression of HSPA5 in hepatocytes [23]. Also it has already been reported that HSPA5 is an antiviral protein for hepatitis A virus replication [24].

In addition, some heat shock proteins helps in the folding of several cellular proteins responsible for the regulation of the cell cycle. Thus, changes in these HSPs may indirectly cause changes in the cell cycles and may be related to tumour formation processes [25, 26].

Because of the important role of heat shock proteins during viral infections, the objective of this study was to identify the changes in the expression profiles of HSPs that occur during HCV infection.

## Materials and methods

### Cell culture and HCV replicon cells

The human hepatoma cell line Huh7.5 was cultured in Dulbecco’s Modified Eagle medium (DMEM) (Sigma-Aldrich, St. Louis, MO, USA) supplemented with 10% foetal bovine serum (Cultilab, Campinas, SP, Brazil), 1% (v/v) non-essential amino acids (Gibco Life Technologies, USA), 100 units/mL of penicillin and 100 μg/mL of streptomycin (Invitrogen, Grand Island, NY, USA) at 37°C in a 5% CO_2_ atmosphere.

In this study we evaluated the interaction of different HSPs on HCV replication using two different genotypes (genotype 2a and genotype 3a). For genotype 2a, two replicon models were used: one presenting the complete genome of HCV (JFH-1) and the other the partial genome of the virus (SGR-Feo-JFH-1). A replicon containing the partial virus genome (S52-SG-Feo) was used to evaluate HCV genotype 3a.

The SGR-Feo-JFH-1 replicon is a bicistronic subgenomic replicon based on the JFH-1 sequence (genotype 2a) that possesses the firefly luciferase gene fused to a neomycin resistance gene and non-structural HCV proteins [27]. The JFH-1 replicon is a full HCV genome construct [28], and second subgenomic replicon used was S52-SG-Feo (genotype 3a) [29].

Huh7.5 cells were electroporated with either SGR-Feo-JFH-1, S52-SG-Feo or JFH-1 RNA. Cells expressing SGR-Feo-JFH-1 and S52-SG-Feo were selected for 21 days with 800 μg/mL of G418 (Sigma-Aldrich, St. Louis, MO, USA). The electroporated JFH-1 cells were cultivated for 17 days after electroporation and were subsequently stored. For all experiments, the cells were plated 24 hours prior to the start of treatments at the following initial cell densities: 1x10^5^ cells/well for a 6-well-plate and 5x10^3^ cell/well for a 96-well-plate. The cell line used in this study was gently provided by Professor Mark Harris of the University of Leeds.

### qPCR array for HSPs

To analyse the gene expression of 84 heat shock proteins, we used a Human Heat Shock Proteins & Chaperones RT^2^ PCR Array (Qiagen), comparing the mock uninfected and infected Huh7.5 cells. Cells were initially lysed in TRIzol reagent and then RNA was isolated using an RNeasy Micro Kit (Qiagen) following the manufacturer′s instructions. Samples were quantified and cDNA synthesis was performed using an RT^2^ First Strand kit (Qiagen). The data were analysed using the ΔΔCt method using the platform provided by Qiagen, available at http://www.sabiosciences.com/pcrarraydataanalysis.php.

### Validation of the qPCR array results

We validated the qPCR array results using a qPCR technique that was performed in three different viral replication models: Huh7.5/JFH-1, Huh7.5/SGR-JFH-1 Feo and Huh7.5/S52. Total RNA was isolated from the cells and then reverse transcribed into cDNA. The samples were used for qPCR using specific, previously synthesized primers. The GAPDH gene was used as an endogenous control. The results were obtained by comparing the relative expression (ΔΔCt) with a standardized cell line without viral replication (Huh7.5).

### RNAi molecules and transfection

To knock down HSPB8 and DNAJC5B gene expression, commercial siRNAs were obtained (EHU160791, Sigma, for HSPB8; and EHU046751, Sigma, for DNAJC5B). As a control, we used a commercial negative control (siRNA#1-NC1) (Applied Biosystems, Foster City, CA, USA) that lacked specificity to any HCV sequence or cellular mRNA used in this study. Different siRNA concentrations (0.1, 1 or 5 nM) were transfected into the cells using Lipofectamine 2000 (Invitrogen, Carlsbad, CA, USA) following the manufacturer’s instructions. The data were analysed after 72 hours of incubation and normalized with mock values (Lipofectamine only).

### Gene expression vectors

To increase the expression of the target genes, the expression vectors pcDNA3.1/HSPB8 (Thermo Fisher Scientific, CA, USA) and pCMV6/DNAJC5B (OriGene Technologies, USA) were used. As controls, the empty expression vectors were used. Approximately 10 μg of each vector was transfected into cells using Lipofectamine 2000 according to the manufacturer′s instructions. The data were analysed after 72 hours of incubation and normalized with mock values (Lipofectamine only).

### Cell viability

The cellular cytotoxicity was determined using an MTT (3-(4,5-dimethylthiazol-2-yl)-2,5-diphenyl tetrazolium bromide) assay (Sigma-Aldrich, St. Louis, MO, USA). Cytotoxicity was assessed in 96-well-plates 72 h after the transfection of cells with siRNAs or vectors. At the end of the incubation, the supernatants were removed and 100 μL of a 1 mg/mL MTT reagent dissolved in FBS-free DMEM was added to the cells. After 30 minutes at 37°C, the MTT solution was removed and 100 μL of DMSO was added (Sigma-Aldrich, St. Louis, MO, USA). The absorption at 562 nm was measured using a spectrophotometer plate reader (FLUOstar Omega/BMG LABTECH, Offenburg, BW, DE).

### RNA isolation and qPCR

Total RNA from transfected cells was isolated using TRIzol reagent (Life Technologies, Carlsbad, CA, USA), and 2 μg of extracted RNA was used for cDNA synthesis using the High-Capacity cDNA Archive Kit (Applied Biosystems, Foster City, CA, USA). The gene expression levels and HCV replication rate (JFH-1) were determined by real-time PCR using Maxima SYBR Green/ROX qPCR Master Mix (Thermo Scientific, CA, USA). GAPDH gene expression was analysed as an endogenous control. Primer sequences are described in the S1 Table. The relative expression values were obtained using the ΔΔCt method.

### Luciferase assay

To assess the level of HCV replication, we evaluated the luciferase activity of Huh7.5 cells stably expressing SGR-Feo-JFH-1 or S52/SG-Feo. Approximately 72 hours after a given treatment, the cells were disrupted with 30 μL of passive lysis buffer (Promega, Madison, WI, USA), and the luciferase substrate (Promega, Madison, WI, USA) was automatically added to each plate well. Luciferase activity was measured using a luminometer (FLUOstar Omega/BMGLABTECH, Offenburg, BW, DE).

### Co-IP and Western blot assays

The IP was performed using Pierce Protein A Agarose (Thermo Fisher Scientific) according to the manufacturer′s instructions. Proteins were extracted from Huh7.5/SGR-Feo-JFH-1 or Huh7.5 S52/SG-Feo cells using Cellytic MT (Sigma-Aldrich, St. Louis, MO, USA) supplemented with protease inhibitor cocktail (Sigma-Aldrich, St. Louis, MO, USA). Cell debris was removed by centrifugation for 10 minutes at 12,000 xg at 4°C. The supernatants were incubated with 10 μL of HCV Antibody NS4B (MA17616) (Thermo Fisher Scientific, CA, USA) overnight at 4°C with stirring. The IP was performed using 200 μL of Pierce Protein A Agarose (Thermo Fisher Scientific) and a 2 hour incubation at room temperature with stirring. The beads were washed (5x) with a buffer containing 25mM Tris and 150mM NaCl, then were resuspended in loading buffer and denatured for 5 minutes at 95°C.

A western blot assay was performed using a 10% denaturing polyacrylamide gel (SDS-PAGE) and the proteins were transferred to a PVDF membrane (Millipore, Bedford, MA, USA). After blocking with 5% (w/v) non-fat milk diluted in TBS-T, the membranes were incubated with the primary antibodies: NS4B (MA17616) (Thermo-Scientific-Pierce, Rockford, IL, USA), at a dilution of 1:3,000; or HSPB8 (OTI1E3) (OriGene Technologies, USA), at a dilution of 1:2,000, or DNAJC5B (PA5-49570) (Thermo-Scientific-Pierce, Rockford, IL, USA), at a dilution 1:200; or GAPDH at a dilution 1:2000 (AM4300) (Thermo-Scientific-Ambion, Rockford, IL, USA) overnight at 4°C. This was followed by an additional incubation with a secondary anti-mouse antibody (HRP) (AB6728) (Abcam, Cambridge, MA, USA) or Anti-Rabbit-HRP (ab6721—Abcam, Cambridge, MA, USA) at a dilution of 1:10,000. The membrane was finally incubated with the Pierce ECL Western Blotting Substrate (Thermo-Scientific-Pierce, Rockford, IL, USA) and luminescence was measured using a Chemi-Doc System (Bio-Rad, Amadora, PT).

### Statistical analysis

The SGR-Feo-JFH-1, JFH-1, S52/SG-Feo expression results and the MTT assay results were calculated as a percentage of the mock sample (medium with Lipofectamine reagent only). All statistical analyses were performed using a one-way ANOVA, followed by Tukey’s post-test using GraphPad Prism 5.0 software (GraphPad Software, San Diego, CA, USA). All experiments were performed in triplicate in three independent events and Standard Deviation (SD) are represented in the graphs or table. The qPCR array was done in 3 independent events. AP value of <0.05 was considered statistically significant.

## Results

### qPCR array results and validation

To evaluate the expression of heat shock proteins in HCV infected cells, a qPCR array experiment was performed. The total RNA from Huh7.5/JFH-1 cells was isolated and reverse transcribed into cDNA. The same procedure was carried out for the uninfected Huh7.5 cell line for comparison. The obtained gene expression values are shown in Table 1, and the 10 genes (listed below) that exhibited higher expression variation in the HCV infected cells are highlighted: CCS, CCT6B, DNAJC12, DNAJC17, DNAJC5B, DNAJC6, HSF4, HSPA6, HSPB6 and HSPB8.

**Table 1:** Analysis of gene expression obtained by the qPCR array for Huh7.5/JFH-1 cells normalized with Huh7.5 cells.

Gene	Expression Log_2_ (SD)	Gene	Expression Log_2_ (SD)	Gene	Expression Log_2_ (SD)
ADCK3	-1.71 (0.26)	DNAJB6	1.05 (0.03)	HSP90AB1	1.13 (0.18)
ATF6	0.95 (1.83)	DNAJB7	0.36 (2.64)	HSP90B1	1.25 (0.04)
BAG1	1.07 (0.04)	DNAJB8	1.42 (0.17)	HSPA14	1.08 (0.08)
BAG2	1.07 (0.01)	DNAJB9	1.30 (1.07)	HSPA1A	1.12 (0.04)
BAG3	1.55 (0.35)	DNAJC1	1.83 (0.57)	HSPA1B	1.15 (0.18)
BAG4	1.14 (0.14)	DNAJC10	1.53 (0.06)	HSPA1L	-1.31 (0.31)
BAG5	1.35 (0.14)	DNAJC11	1.14 (0.17)	HSPA2	-1.14 (0.12)
**CCS**	**1.87 (0.08)**	**DNAJC12**	**2.49 (0.26)**	HSPA4	1.23 (0.07)
CCT2	-1.05 (0.05)	DNAJC13	1.82 (0.15)	HSPA4L	1.62 (0.03
CCT3	-1.29 (0.33)	DNAJC14	1.07 (0.06)	HSPA5	1.26 (0.10)
CCT4	-1.28 (0.32)	DNAJC15	1.24 (0.02)	**HSPA6**	**2.03 (0.43)**
CCT5	1.07 (0.01)	DNAJC16	1.22 (0.15)	HSPA8	(1.05 (0.06)
CCT6A	-1.22 (0.09)	**DNAJC17**	**4.11 (1.12)**	HSPA9	1.54 (0.05)
**CCT6B**	**1.94 (0.13)**	DNAJC18	1.48 (0.19)	HSPB1	1.55 (0.35)
CCT7	-1.08 (0.05)	DNAJC21	1.28 (0.05)	HSPB2	Undetermined
CRYAA	Undetermined	DNAJC3	1.82 (0.37)	HSPB3	Undetermined
CRYAB	1.79 (0.00)	DNAJC4	1.12 (0.09)	**HSPB6**	**1.96 (0.30)**
DNAJA1	1.09 (0.03)	DNAJC5	-1.19 (0.18)	HSPB7	1.24 (0.02)
DNAJA2	1.18 (0.06)	**DNAJC5B**	**3.27 (1.82)**	**HSPB8**	**4.18 (1.13)**
DNAJA3	1.20 (0.13)	DNAJC5G	Undetermined	HSPD1	1.09 (0.06)
DNAJA4	-1.48 (0.52)	**DNAJC6**	**1.85 (0.02)**	HSPE1	-1.51 (0.76)
DNAJB1	1.26 (0.18)	DNAJC7	1.19 (0.02)	HSPH1	1.37 (0.02)
DNAJB11	1.18 (0.05)	DNAJC8	1.39 (0.10)	PFDN1	1.20 (0.09)
DNAJB12	1.05 (0.03)	DNAJC9	-1.27 (0.31)	PFDN2	1.11 (0.06)
DNAJB13	-1.38 (0.48)	HSF1	1.18 (0.07)	SERPINH1	-1.77 (0.29)
DNAJB14	1.72 (0.05)	HSF2	1.28 (0.03)	SIL1	1.15 (0.12)
DNAJB2	1.82 (0.16)	**HSF4**	**1.9 (0.22)**	TCP1	-1.16 (0.17)
DNAJB5	1.50 (0.31)	HSP90AA1	1.10 (0.09)	TOR1A	1.25 (0.04)

**Fig 1:**
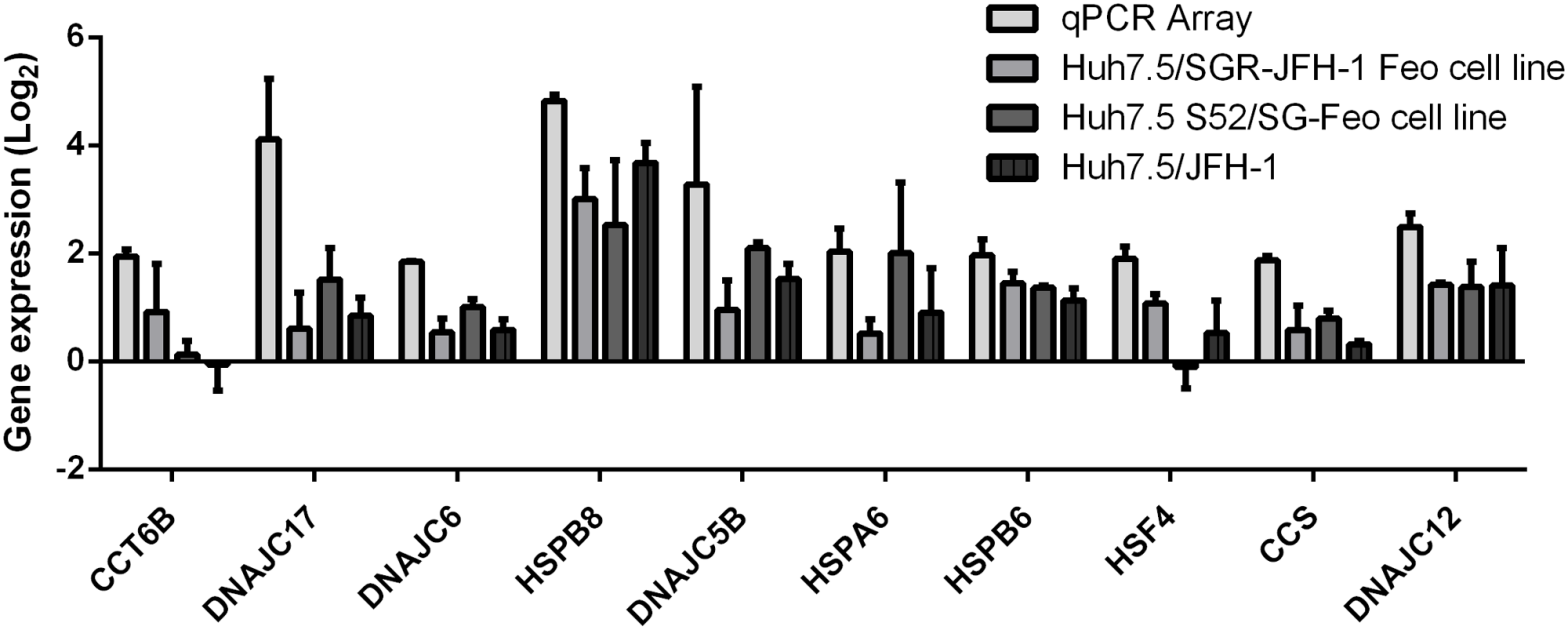
HSPs array. Analysis of the qPCR array HSPs, showing 10 genes that showed the highest increase in gene expression and validated with SGR-JFH-1 Feo, JFH-1 and S52/SG-Feo viral models. The values were normalized using Huh7.5 cells.

To validate these results, specific primers were designed for the genes highlighted in Table 1. In addition to the viral model Huh7.5/JFH-1, two other models (SGR-JFH-1 Feo andS52/SG-Feo cell lines) were used to confirm the observed differences in the expression of HSPs. The results of the gene expression analysis were plotted on a graph in Log_10_ scale (Fig 1).

We adopted an arbitrary value of ≥2 as a cut off [30]. Using this criterion three genes were validated as having altered expression. The HSPB8 gene (Heat Shock Protein Family B Member 8) was overexpressed in both genotypes evaluated. JFH-1 and SGR-JFH-1-Feo virus models for genotype 2a and model S52 / SG-Feo for genotype 3a.

The DNAJC5B (DnaJ Heat Shock Protein Family Member C5 Beta) and HSPA6 (Heat Shock Protein Family A Member 6) genes were overexpressed for HCV genotype 3a (model S52 / SG-Feo).

It was previously shown that the HSPA6 protein interacts with the 3′ NCR region of HCV; therefore, we chose to explore the other two validated genes [31, 32].

### The role of the HSPB8 gene in HCV replication

**Fig 2:**
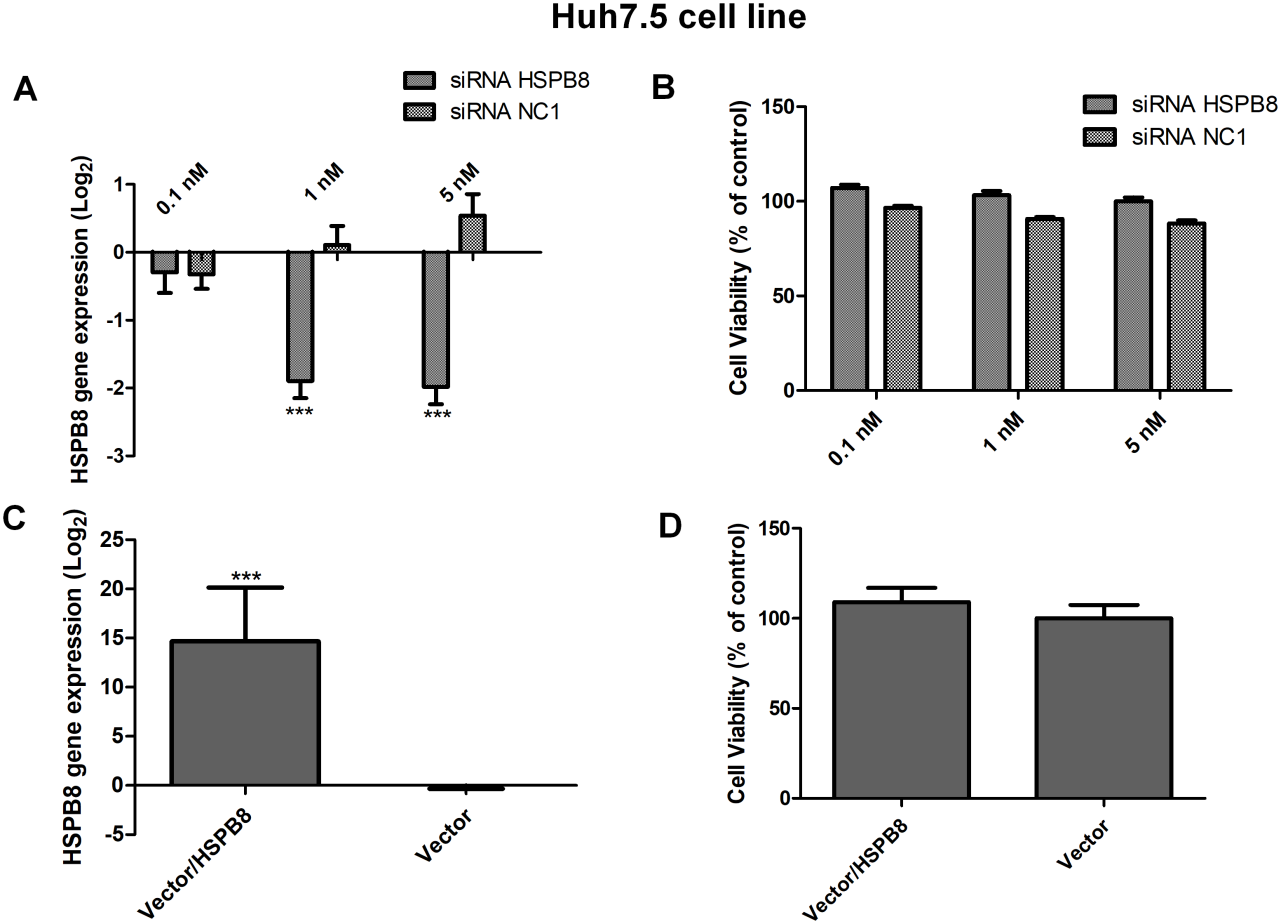
HSPB8 gene expression and cell viability using the Huh7.5 cell line. A: Determination of HSPB8 gene expression by qPCR in a cell line transfected with siRNA for HSPB8 at concentrations of 0.1, 1 or 5 nM. B: Determination of the cell viability of Huh7.5 cells when transfected with siRNA for HSPB8 at three concentrations. A scrambled siRNA (NC1) was used as a control. C: Determination of HSPB8 gene expression by qPCR in a cell line transfected with the HSPB8 vector. D: Determination of cell viability by an MTT assay in a cell line transfected with the HSPB8 vector. An empty vector was used as a control. All assays were performed at 72 hours post-transfection and the data were normalized with mock values (transfection reagent only). ***P<0.0001 vs. Mock.

The HSPB8 gene showed an increase in gene expression in all the tested viral models, confirming the results obtained in the qPCR array experiment. To better understand the role of HSPB8 in HCV replication, we inhibited the expression of this gene using specific siRNA molecules. First, we evaluated its gene inhibition cell and toxicity by qPCR (Fig 2A) and an MTT cell viability assay, respectively (Fig 2B). The three siRNA concentrations tested did not significantly alter cell viability, and concentrations of 1 and 5 nM silenced the HSPB8 gene. Protein expression of HSPB8 was also confirmed by Western Blot (S1 Fig).

**Fig 3:**
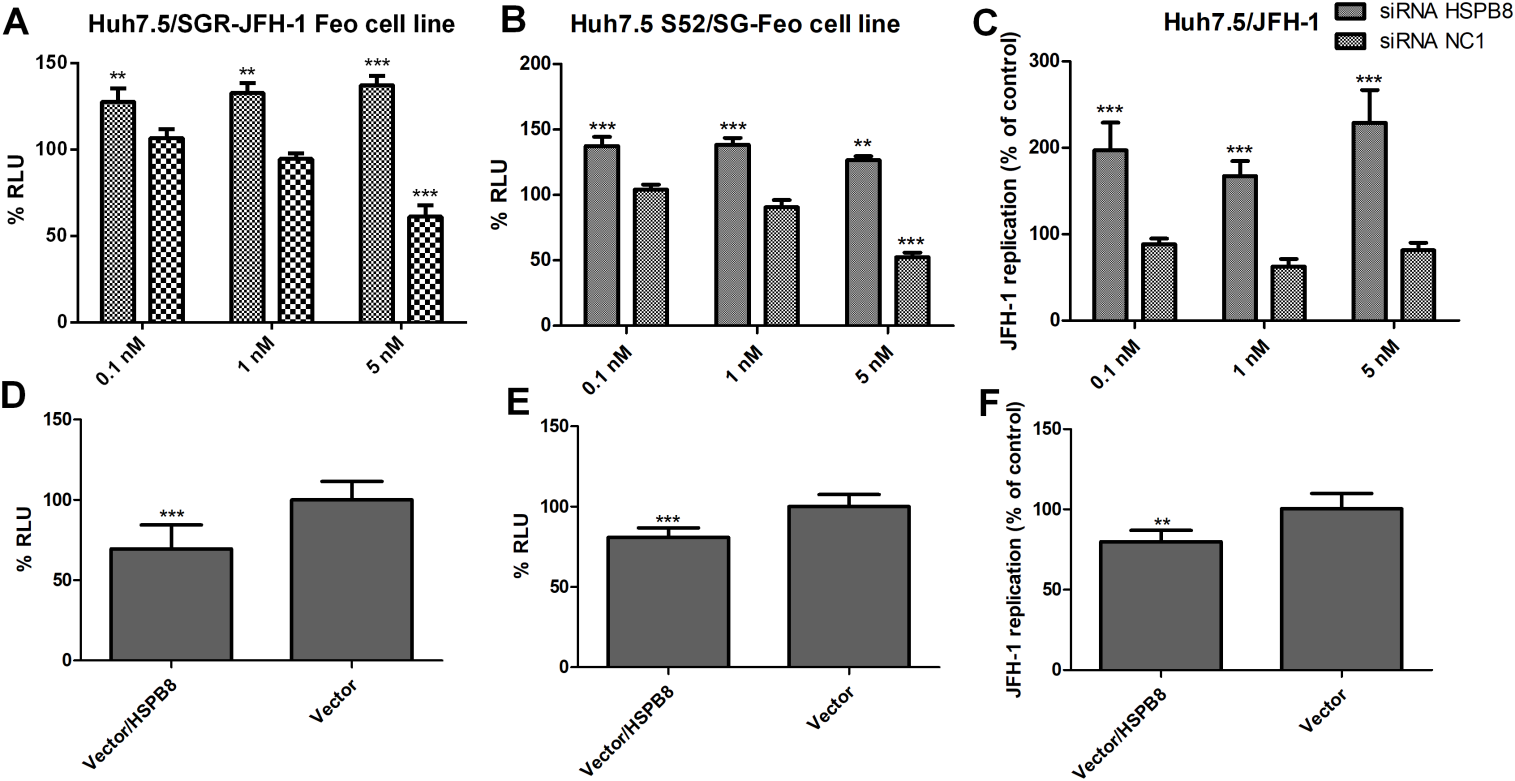
HCV replication in three different viral models. A: Determination of HCV replication by a luciferase assay using Huh7.5/SGR-JFH-1 Feo cells treated with siRNA HSPB8 and a negative control (siRNA NC1) at concentrations of 0.1, 1 and 5 nM. B: Determination of HCV replication by a luciferase assay using Huh7.5 S52/SG-Feo cells. C: Determination of HCV replication by qPCR using Huh7.5/JFH-1 cells. D: Determination of HCV replication by a luciferase assay using Huh7.5/SGR-JFH-1 Feo cells treated with vector/HSPB8 or empty vector. E: Determination of HCV replication by a luciferase assay using Huh7.5 S52/SG-Feo cells. F: Determination of HCV replication by qPCR usingHuh7.5/JFH-1 cells. All assays were performed at 72 hours post-transfection and the data were normalized with mock values (transfection reagent only). ***P<0.0001 vs. Mock.

The cell lines expressing viral replicons were transfected with siRNA targeting HSPB8 mRNA. When the translation of this cellular protein was inhibited, we observed a greater than 20% increase in viral replication in all analysed viral models (Fig 3A–3C). In the subgenomic models, 5 nM of the control siRNA significantly altered HCV replication, therefore the data generated using this concentration were not considered (Fig 3A and 3B).

To confirm the observed effect on viral replication, we performed the reverse procedure by increasing the expression of the HSPB8 gene using an expression vector. First, we confirmed the increase in the target gene expression (Fig 3C) and assessed cell viability (Fig 3D). We then proceeded to increase the expression of HSPB8 in all three viral models and in all cases observed at least a 20% decrease in viral replication relative to the control (empty vector) (Fig 3D and 3F).

### The role of the DNAJC5B gene in HCV replication

**Fig 4:**
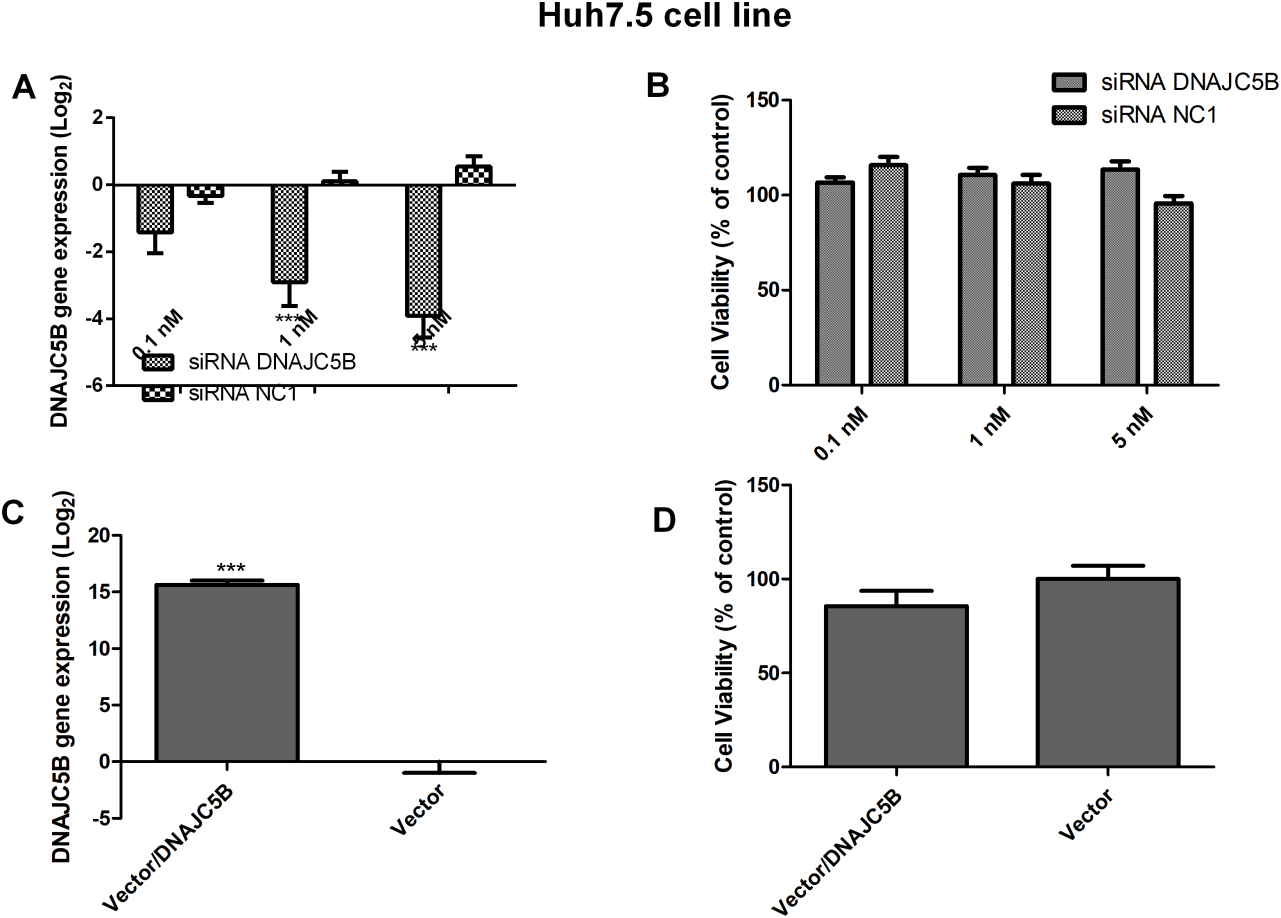
DNAJC5B gene expression and cell viability of the Huh7.5 cell line. A: Determination of HSPB8 gene expression by qPCR in a cell line transfected with siRNA for DNAJC5B at concentrations of 0.1, 1 or 5 nM. B: Determination of cell viability by an MTT assay in a cell line transfected with siRNA for HSPB8 at three concentrations. As a control, a scrambled siRNA (NC1) was used. C: Determination of DNAJC5B gene expression by qPCR in a cell line transfected with the DNAJC5B vector. D: Determination of cell viability by an MTT assay in a cell line transfected with the DNAJC5B vector. As a control, an empty vector was used. All assays were performed at 72 hours post-transfection and the data were normalized with mock values (transfection reagent only). ***P<0.0001 vs. Mock.

Because the altered DNAJC5B was validated in genotype 3a HCV strain (S52), this gene was further evaluated. The cell viability assay and confirmation of gene inhibition are shown in Fig 4A and 4B. At an siRNA concentration of 1 nM, silencing of the target gene was observed. In addition, the siRNA molecule was capable of altering the viability of the cells.

**Fig 5:**
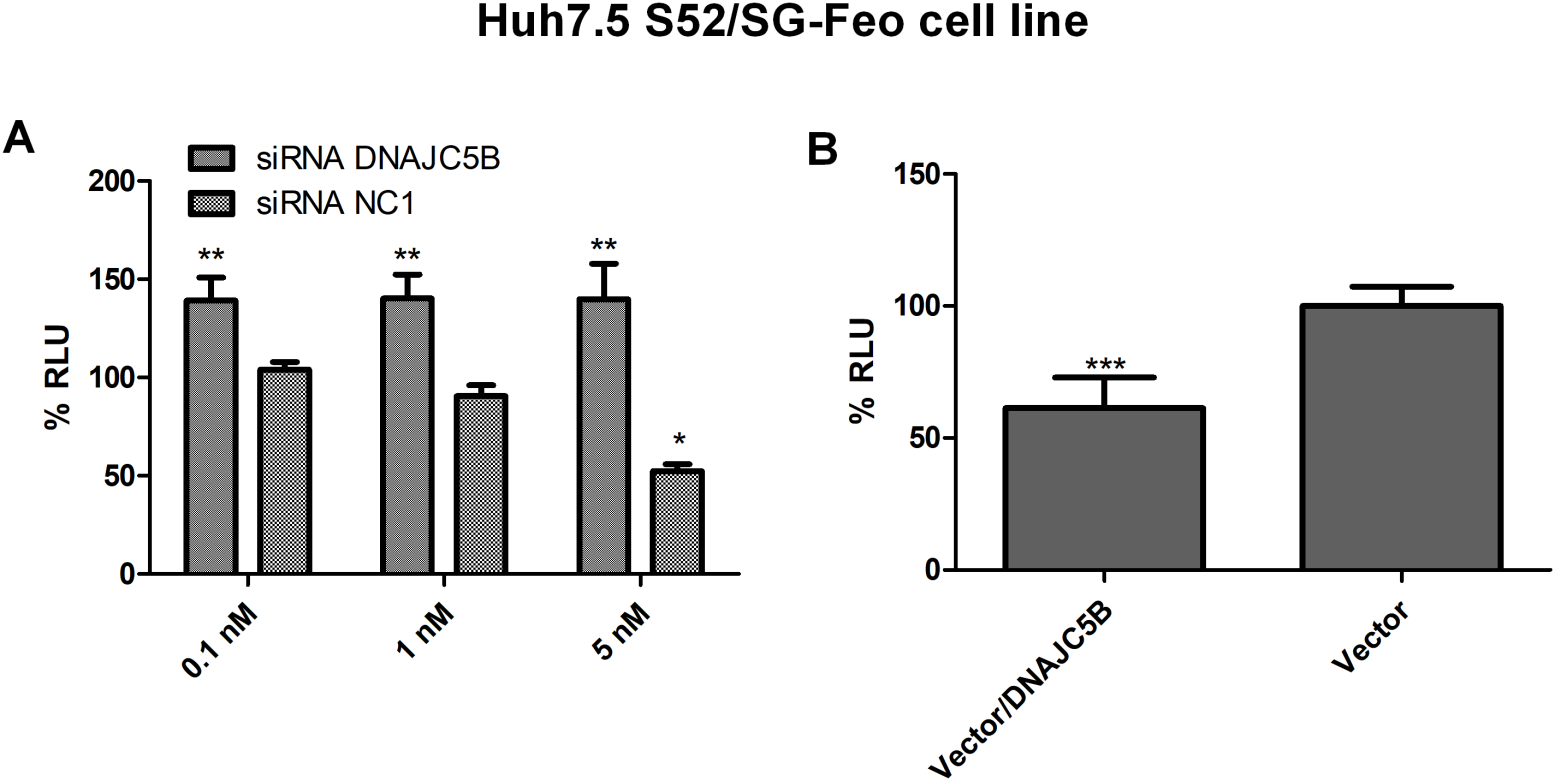
HCV replication in genotype 3 viral models. A: Determination of HCV replication by a luciferase assay with Huh7.5 S52/SG-Feo cells treated with siRNA DNAJC5B or a negative control (siRNA NC1) at concentrations of 0.1, 1 or 5 nM. B: Determination of HCV replication by a luciferase assay with S52/SG-Feo cells treated with vector/DNAJC5B or empty vector. All assays were performed at 72 hours post-transfection and the data were normalized with mock values (transfection reagent only). ***P<0.0001 vs. Mock.

After confirming the efficiency of DNAJC5B siRNA molecule and that gene silencing did not affect cell viability, a transfection of this siRNA was performed with the Huh7.5 S52/SG-Feo cell line. The results showed an increase in viral replication when this cellular protein exhibited reduced expression (Fig 5A).

To confirm the role of this protein in HCV replication, we also increased the expression of DNAJC5B. The gene expression and cell viability values are shown in Fig 4C and 4D. The expression of HCV, genotype 3a, was reduced by more than 40% when DNAJC5B was overexpressed (Fig 5B).

### Protein-protein interaction

**Fig 6:**
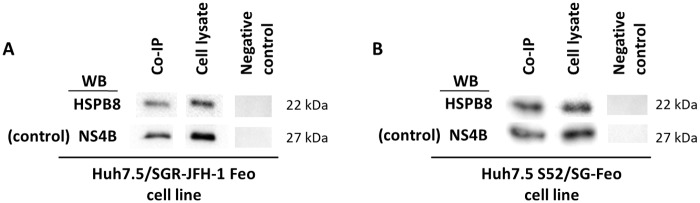
Co-immunoprecipitation assay (Co-IP). The NS4B viral protein was immunoprecipitated from cell lysate with a specific antibody and Protein A agarose. As a negative control, the immunoprecipitation reaction was performed without an antibody to NS4B. WB: Western blot detection of specific proteins NS4B (Co-IP control) and HSPB8. A: Assay performed on the Huh7.5/SGR-JFH-1 Feo cell line. B: The assay performed on the Huh7.5 S52/SG-Feo cell line.

Because the HSPB8 protein showed good results in all the tested viral models, we evaluated its interaction with the viral protein NS4B, which acts at several stages of HCV replication. To determine whether the two proteins directly interact, we performed a co-immunoprecipitation assay. The NS4B viral protein was precipitated by a specific antibody and a western blot assay was subsequently performed, resulting in HSPB8 being detected. This phenomenon was observed for both HCV genotypes (Fig 6A and 6B).

## Discussion

Hepatitis C virus infection modulates the expression of several cellular proteins to promote viral replication. In our study, we observed that HCV alters the expression of many heat shock proteins and chaperones. The increased expression of the HSPB8 and DNAJC5B genes observed in the qPCR array indicated that these proteins either promote HCV replication or were expressed by the cells due to stress resulting from the viral infection.

The validation of the HSPB8 gene in all tested viral models (for both genotype 2a and genotype 3) is an interesting result since it has a similar expression profile for several genotypes, suggesting a more global cellular mechanism. Previous studies have shown that the HSPB8 protein is more highly expressed in HCV-infected Huh7.5 cells, corroborating our results [33].

HSPB8 silencing promoted an increase in viral replication in all models tested and increased HSPB8 gene expression led to an opposite effect (a reduction of HCV). These results suggest that this protein is expressed as a form of cellular defence against viral infection and that HSPB8 can decrease the replication of HCV through a mechanism that is not yet understood.

There are no data that links the HSPB8 protein to a cellular defence mechanism for viral infections. However, this function has been described for HSP27, another protein belonging to the small heat shock protein family. Studies have shown that when HSP27 is inhibited by siRNA, a nearly two-fold increase in Hepatitis B virus (HBV) production was observed when compared to control cells. In addition, when HSP27 was overexpressed, a decrease in HBV production was observed, suggesting that HSP27 functions as an endogenous anti-HBV factor. The study further demonstrated that HSP27 expression activated type I IFN [34].

In this study, we showed, for the first time, that HSPB8 directly interacts with the viral protein NS4B, which participates in several stages of HCV replication. Previous studies had shown that NS4B interacts with others HSPs, such as HSP70, HSPA8 and HSP90B1 [35, 36].

The higher expression of DNAJC5B, also known as the CSP-ß gene, was more expressive in the Huh7.5 S52/SG Feo strain (genotype 3) than in genotype 2. Its inhibition by RNAi also promoted an increase in viral replication and its overexpression promoted a viral reduction, suggesting that it is a cellular protection factor. However, the occurrence of this phenomenon specifically in genotype 3 may aid in the elucidation of some factors that are observed only in this genotype, such as the increase of hepatic steatosis, insulin resistance, the differential response to medication and the occurrence of viral resistance [37–39]. DNAJC5B may be acting on the pathways that the HCV genotype 3 alters. This protein is poorly studied, although some studies have reported that tissues, such as the testis and brain, have a higher expression of DNAJC5B, and it probably acts within several secretory pathways [40, 41].

In our study, we observed that the increase in the expression of HSPs does not promote viral replication but rather is a possible cellular defence mechanism to reduce viral replication. The mechanisms involved in this process, and the possible interactions of these chaperones with viral proteins, remain to be elucidated.

## Supporting information

S1 Table Sequences of PCR primers used in this study. (DOCX)

S1 Fig Western blot assay performed on Huh7.5 cells 72 hours after transfection of siRNA molecules or expression vectors. A: Protein expression of HSPB8. B: Protein expression of DBAJC5B. GAPDH was used as an endogenous control.
(PDF)
