# Supplementary material for: Heat shock proteins HSPB8 and DNAJC5B have HCV antiviral activity
Source: PLoS One. 2017 Nov 28;12(11):e0188467. doi: 10.1371/journal.pone.0188467 (PMC5705118; doi:10.1371/journal.pone.0188467)
Supplement: S1 Table — (DOCX) [file pone.0188467.s001.docx]

**S1 Table**

| **Gene** | **Sequence 5'-3'** | **Size** |
| --- | --- | --- |
| **CCS** | F - TCCTACAGCTGACCCCTGAG | 99 |
|  | R - TAAGGTCCCCGTACTGATGG |  |
| **DNAJC5B** | F - AAACGGTGGAACAGTTTTGC | 120 |
|  | R - GCTCCCTTATGCAGACCAAG |  |
| **DNAJC12** | F - GTTTCCGAGGGAAGAAGGAC | 133 |
|  | R - CTGCCAGGATTTGTTCAACC |  |
| **DNAJC17** | F - GCCTGAGAGAAGAGGGTTCC | 101 |
|  | R - TTCTGCCTTTCCTCTCAACC |  |
| **HSPA6** | F - TTGACGCTGGTGTCTTTGAG | 94 |
|  | R - TCCATGAAGTGGTTCACGAG |  |
| **HSPB6** | F - TCTCACCCACTCCTGGATTC | 149 |
|  | R - GTGCTGGTAGGGTCTGGAAG |  |
| **HSPB8** | F - TGTCCAGCACGTTCTCAAAG | 105 |
|  | R - CACAGGGGACAGAAGAAAGG |  |
| **CCT6B** | F - TCTTCGCAGAGCAAAAAGAAG | 107 |
|  | R - CAGCATGTCCCAAGCAATC |  |
| **DNAJC6** | F - TGGATTCCAGACATCTTGACC | 148 |
|  | R - TTCCGACACACAGCAAAAAG |  |
| **HSF4** | F - GTTTCCTCGTAAGCGACCAG | 111 |
|  | R - TCCGAAAACCGTACATGTTG |  |
| **HCV** | F - CTCCGCCATGAATCACTC | 120 |
|  | R - ACGACACTCATACTAACGC |  |
