# Supplementary figures and images for: Heat shock proteins HSPB8 and DNAJC5B have HCV antiviral activity
Source: PLoS One. 2017 Nov 28;12(11):e0188467. doi: 10.1371/journal.pone.0188467 (PMC5705118; doi:10.1371/journal.pone.0188467)

S1 Fig

**A**

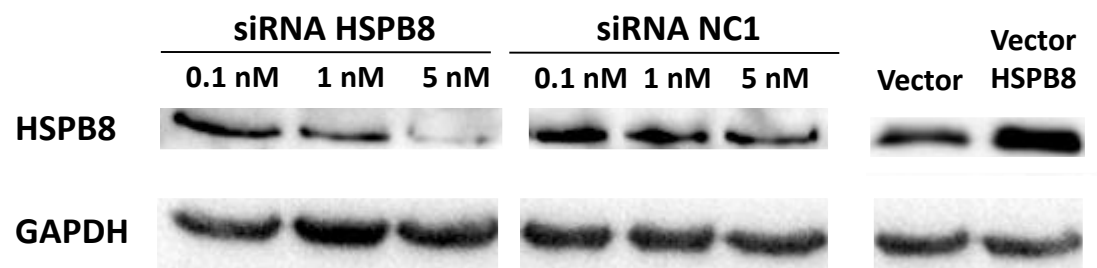

**B**

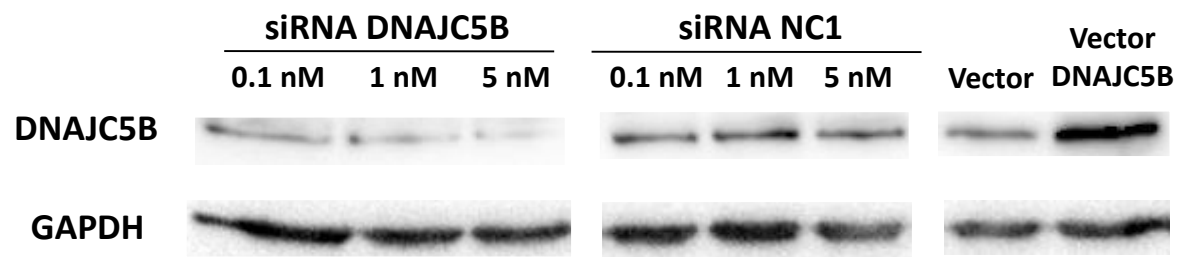

Supplement: S1 Fig — A: Protein expression of HSPB8. B: Protein expression of DBAJC5B. GAPDH was used as an endogenous control. (PDF) [file pone.0188467.s002.pdf]
